# Multidimensional Scale of Perceived Social Support in Indonesian adolescent disaster survivors: A psychometric evaluation

**DOI:** 10.1371/journal.pone.0229958

**Published:** 2020-03-13

**Authors:** Okki Dhona Laksmita, Min-Huey Chung, Yuan-Mei Liao, Pi-Chen Chang

**Affiliations:** 1 School of Nursing, College of Nursing, Taipei Medical University, Taipei, Taiwan; 2 Department of Nursing, Taipei Medical University-Shuang Ho Hospital, New Taipei City, Taiwan; 3 Institute of Clinical Nursing, College of Nursing, National Yang-Ming University, Taipei, Taiwan; University of Ghana, GHANA

## Abstract

**Background:**

Social support plays an important role in adolescents’ mental health and well-being, and even more so for disaster survivors. To measure the level of social support, one needs an appropriate tool to produce valid and reliable results; therefore, we aimed to measure the invariance across gender groups, and analyze the construct validity and reliability of the Indonesian version of the Multidimensional Scale of Perceived Social Support (MSPSS), a social support measurement tool which was theoretically constructed and has been well validated in many countries with various cultures and backgrounds.

**Methods:**

A school-based assessment was conducted in junior and senior high schools in a post-disaster setting in Yogyakarta Province, Indonesia. We analyzed 299 adolescent survivors of a volcanic eruption, aged 12~18 years who completed a 12-item Indonesian version of the MSPSS.

**Results:**

The factorial validity confirmed the three-factor structure of the scale (Family, Friends, and Significant Others) which met all of the criteria of parameter indices and provided evidence of high internal consistency reliability. The three-level measurement of invariance, which consisted of configural, metric, and scalar invariance, also performed very well across gender groups with our data and corresponded to the recommended parameters. Our composite reliability values were all fine (>0.7) and indicated that the items in the same construct were strongly correlated and reliable.

**Conclusions:**

The Indonesian version of the MSPSS was shown to be a valid, reliable, theoretically constructed, and applicable instrument for adolescent disaster survivors.

## Introduction

Disasters have been reported to have long-term impacts on vulnerable groups, such as children and adolescents [1–3], and they deserve our attention. Vulnerability is indicated by a transition period from childhood to adulthood when they are physically and psychologically growing, and a disaster would definitely make them to more vulnerable. Social support is often disrupted in such populations after a disaster [4–6]. At the same time, social support plays an important role as a buffer against negative effects of disaster-related traumatic events. This protective effect of social support can help them recover from the event [2, 6, 7] and minimizes the potential of developing depression and posttraumatic stress disorder (PTSD) symptoms [8–11]. Therefore, evaluating social support among this population is very important and must be appropriately assessed using a robust, valid, and reliable measurement tool.

The Multidimensional Scale of Perceived Social Support (MSPSS) is a frequently used scale [12] and has been adapted by many studies in multicultural settings and diverse populations [13–16]. It has a three-factor structure which measures Family, Friends, and Significant Others. Social support from these groups is very meaningful for adolescents, and it gives them a sense that support is reliable and available when they need it [13, 17]. It was initially developed in US undergraduate students aged 17~22 years old [13] followed by other studies which confirmed the factorial validity of a three-factor structure for US adolescents and adults [17, 18], Turkish adults [19], Singaporean adults [20], and Chinese adults [21] and of a two-factor structure for Chinese adolescents [16], Hispanic adult immigrants [22], US older adults [23], and South Asian adults [24]. However, testing of the scale’s validity in disaster survivors is lacking, particularly in Indonesian adolescent populations. No psychometric evaluation of the MSPSS for use among Indonesian populations has been conducted. The results of our study can be used to develop social support research and interventions for Indonesian adolescent disaster survivors.

The literature suggests that gender differences exist in social support scales. However, there is a need to ensure the measurement invariance [21, 25]. Thus, it is very important to provide a prerequisite for meaningful comparisons across groups because measurement invariance examines whether populations of different genders interpret a given measure in a conceptually similar manner over several performance dimensions [26]. Measuring social support using a reliable instrument that produces valid research data across different groups of adolescent disaster survivors would be of critical importance in moving this research forward. Therefore, our purpose in this study was to establish valid comparisons of MSPSS item responses and its subscales across specific groups in Indonesia to confirm the construct validity and measurement invariance.

## Materials and methods

### Study design, setting and participants

A cross-sectional study design was applied in this study, and a stratified sampling method were used to select participants. The median sample size in Structural Equation Modeling is about 200 cases according to reviews of studies in different settings and disciplines [27, 28]; therefore, on September 2017 we invited the participation of 310 students who had experienced the 2010 Mount Merapi eruption, aged 12~18 years, and who study at schools (four senior and three junior high schools) in a post-volcanic eruption setting in Cangkringan District, the district most severely damaged by the eruption in Yogyakarta Province [29]. We distributed a parental consent form to be signed by the parents or guardian at home and also a student assent form to be signed by participants. The following day, participants who returned a completed parental consent and student assent form were then requested to complete a social support scale in their classrooms. Among the 310 participants, 299 of them met the inclusion criteria.

### Instrument

Permission to use the MSPSS and its Indonesian version was granted by the original author [13] and translator [30, 31]. The copyright of the original version belongs to the original author [13], and that of the Indonesian version belongs to the translator [30, 31]. A reproduction of the scale was only obtained from those publishers. The MSPSS is a brief 12-item, self-administered measurement tool with three subscales: Family (items 3, 4, 8, and 11), Friends (items 6, 7, 9, and 12), and Significant Others (items 1, 2, 5, and 10). Every item uses a seven-point Likert scale ranging from 1 (very strongly disagree) to 7 (very strongly agree). A higher score indicates greater the social support perceived by an individual; the total possible score ranges 12~84, or it can be scored according to its subscales by adding the items in each subscale and then dividing by 4. Additionally, the scale is very easy to administer and is user friendly for both the administrator and participants [13, 15]. We tested the reading level of the Indonesian version for its face validity in 48 students from seventh to ninth grades, and it was found that all the words used in the scale were understandable.

The original version of the MSPSS has very good internal reliability with an α coefficient of 0.88 for the total scale, 0.87 for the Family subscale, 0.85 for the Friends subscale, and 0.91 for the Significant Others subscale [13]. In addition, its test-retest reliabilities after 2~3 months from the initial data collection were 0.85, 0.75, 0.72, and 0.85 for the Family, Friends, and Significant Others subscales, and overall scale, respectively. The construct validity between the scale and depression scores yielded an inverse correlation with an *r* value of -0.25.

The Indonesian version was translated using guidelines from Brislin [32] and first validated in 18~65-year-old Indonesian family caregivers of schizophrenia patients. The reliability value of the translated scale was 0.85, and the content validity index for the scale (S-CVI) of each content relevance and clarity was 1 [30, 31].

### Data analysis

This study used statistical software of IBM SPSS version 21.0 and IBM SPSS for AMOS version 21.0 to analyze the data. We investigated participants’ demographic characteristics using a descriptive statistical analysis. No missing data were found in the analysis. We then replicated the original three-factor structure of the original MSPSS for use in our present study and examined whether the three-factor structure of the Indonesian version was consistent with the original one. We conducted a confirmatory factor analysis (CFA) because we wanted to assign the three factors (Family, Friends, and Significant Others) as a latent construct and measure each of them using four directly measurable items. This analysis determined the goodness of fit between the observed data and the hypothesized model through performing a first-order CFA with some model fit parameters including the ratio of the Chi-squared value to the degree of freedom (*x*^*2*^*/df*), root-mean-square error approximation (RMSEA), goodness of fit index (GFI), comparative fit index (CFI), Tucker-Lewis index [33], and standardized root mean square residual (SRMR). An acceptable goodness of fit of the model was determined by *x*^*2*^/*df* <5, RMSEA <0.08, GFI >0.90, CFI >0.90, TLI >0.90, and SRMR <0.08 as recommended by previous studies [34, 35].

We also investigated more details about the scale’s characteristics by evaluating three levels of the measurement invariance; the same construct was similarly measured across groups, and consisted of configural, metric, and scalar invariances across gender groups through a multi-group (MG)-CFA [36]. The MG-CFA is a very common procedure for measuring equivalence across groups, which are compared in terms of factor analytic parameters such as factor loadings, intercepts, error variances, factor variances, covariances, and factor means [26, 37, 38]. The first level, configural invariance [39], assumes that the same pattern of item-factor loadings exists across the groups being compared. It also suggests that the same items must have nonzero loadings on the same factors. Additionally, configural invariance demonstrates the same number of factors in every group, which then determines the requirement for the other tests. The second level, metric invariance [40], investigates the factor loadings’ invariance across groups. The last, scalar invariance which is the strongest level measurement of invariance [39, 41], examines the item intercepts across groups. However, scalar invariance must meet the assumptions of both configural and metric invariances.

We referred to some references as guides to perform the analysis and indices for tests of invariance. Most studies using the MG-CFA approach compared the fit of a baseline model to the fit of an increasingly constrained model. We employed the indices recommended by Cheung and Rensvold [42] and French and Finch [37] using differences in the CFI (ΔCFI) and the RMSEA (ΔRMSEA) for which values of ≤0.01 indicate strong invariance [37, 42].

In addition, we conducted a reliability test to evaluate the within-scale consistency of the responses to the items of the measure. We used Cronbach’s α (CA) and the composite reliability (CR) as the internal consistency of each factor [43]. CA and the CR are two different calculations, but may prove the same thing, although they might not have the same value. The initial method used to measure the reliability was CA; however, several researchers have argued that it has some limitations [44], i.e., it may over- or underestimate reliability [43, 45], and furthermore, underestimation of the true reliability may become a problem when the test is multidimensional. It might not be appropriate to use CA as an estimate of the reliability of a multidimensional composite scale score, even though the correlation between dimensions is strong [46]. In addition, the assumption of parallelity of CA indicates that all factor loadings and error variances are equally constrained. Hence, the CR is a better choice to measure the reliability due to its ability to draw on standardized regression weights and measurement correlation errors for each item, and a value of >0.7 is acceptable [47–49].

## Results

### Participant characteristics

In total, 310 questionnaires were distributed, and 299 (96.5%) valid questionnaires were analyzed. Table 1 demonstrates the participants’ characteristics.

**Table 1:** Participants’ demographic characteristics.

Variable	*N* = 299	
	*n*	%
Age (years old) (mean±SD = 15.02±1.75)		
12~15	160	53.51
15~18	139	46.49
Gender		
Male	143	47.83
Female	156	52.17
Grade in school		
Junior high	104	34.78
Senior high	195	65.22
School		
Private	93	31.10
Public	206	68.90
Residency		
Cangkringan District	162	54.18
Others	137	45.82
Religion		
Muslim	294	98.33
Non-Muslim	5	1.67
Living arrangement		
With parents	278	92.98
With others	21	7.02

SD, standard deviation

### Confirmatory factor analysis (CFA)

**Fig 1:**
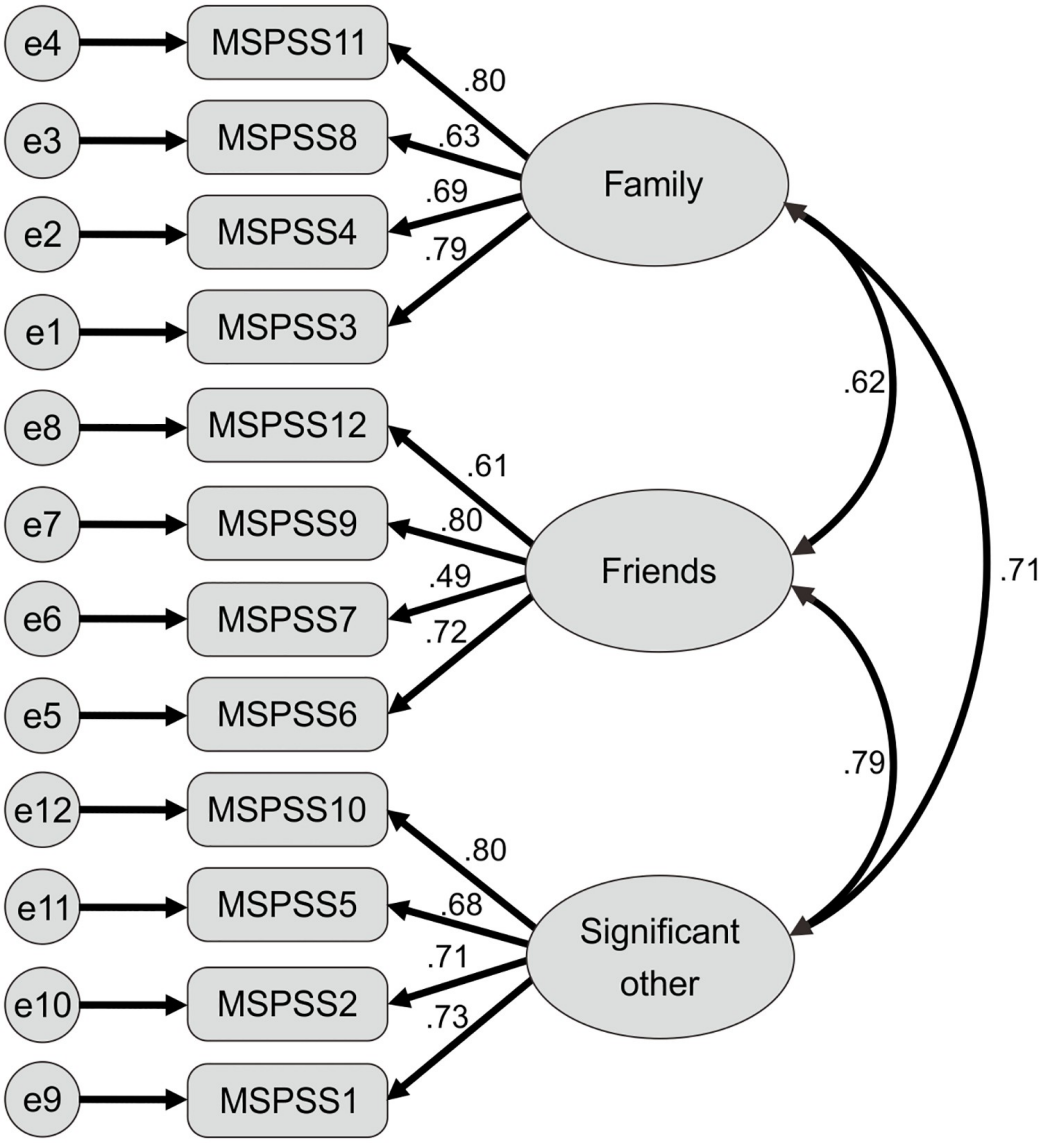
The three-structure model of the Indonesian version of the Multidimensional Scale of Perceived Social Support (MSPSS).

Results of the CFA indicated that the hypothesized three-factor structure demonstrated a very good fit to the data, because all of the parameters corresponded to the recommended criteria very well. The results indicated that *x*^*2*^/*df* = 2.468, RMSEA = 0.070, GFI = 0.935, CFI = 0.948, TLI = 0.933, and SRMR = 0.047. Using standardized estimates, our factor loadings ranged 0.49~0.80, and were significant (Fig 1). The results met the recommended criteria from Hooper, Coughlan (34).

### Measurement invariance

#### Configural invariance

Configural invariance was examined to determine whether the Indonesian version of the MSPSS was best described by a three-factor structure across the gender groups. Configural invariance was good (as evidenced by good model fit measures when freely estimating two groups, i.e., without constraints). The statistical analysis demonstrated that the configural invariance model (M1) fit the data very well with CFI = 0.931, RMSEA = 0.057, and all factor loadings being significant (*p*<0.05). The two groups were equivalent (Table 2).

**Table 2:** Fit indices for measurement invariances across genders.

Model	*x*^*2*^	*df*	*x*^*2*^*/df*	RMSEA	CFI	TLI	SRMR	Comparison	ΔCFI	ΔRMSEA
M0	125.884	51	2.468	0.070	0.948	0.933	0.0473			
M1	200.703	102	1.968	0.057	0.931	0.910	0.0541			
M2	223.178	114	1.958	0.057	0.923	0.911	0.0594	M2 vs. M1	0.008	0.000
M3	256.493	126	2.036	0.059	0.908	0.904	0.0577	M3 vs. M2	0.015	0.002

*x*^*2*^*/df*, ratio of Chi-squared to the degrees of freedom; RMSEA, root mean square error approximation; CFI, comparative fit index; TLI, Tucker-Lewis index; SRMR, standardized root mean square residual; M0, initial model; M1, configural invariance model (unconstrained/ baseline model); M2, metric invariance model (fully constrained); M3, scalar invariance model.

#### Metric invariance

A metric invariance model (M2) was also investigated to explore the invariance of factor loadings between the two groups. The model described a very good fit with CFI = 0.923 and RMSEA = 0.057. With regard to the ΔCFI and ΔRMSEA, significant changes occurred; ΔCFI = 0.008 and ΔRMSEA = 0.000 (Table 2).

#### Scalar invariance

The last measurement invariance, a scalar analysis, was carried out to examine whether the intercepts and factor loadings were equal between the two groups. The model (M3) investigated if the factor loadings and intercepts were all constrained to be equal across groups, and the residual variances were freely estimated. The analytical results describing the data fit very well; CFI = 0.908 and RMSEA = 0.059. ΔCFI and ΔRMSEA values were 0.015 and 0.002, respectively, both of which were acceptable (Table 2).

### Reliability

To assess the internal consistency reliability, CA and the CR were calculated for the total Indonesian version of the MSPSS and for each subscale. Results are presented in Table 2 in which coefficients for both the 12 items of the scale and across gender groups were considered acceptable (α >0.70) [50]; hence, results of our study met the recommended criteria. In addition, CR values ranged 0.74~0.83, indicating that the internal consistencies of these values were adequate, because they corresponded well with the suggested level of >0.70 [47, 48]. The literature states that CR values of > 0.70 indicate that items in the same construct are strongly correlated, and that all items are reliable [47] (Table 3).

**Table 3:** Multidimensional Scale of Perceived Social Support item distributions and their characteristics.

Item number and description	Mean±SD	Cronbach’s α			Composite reliability		
		Overall	Male	Female	Overall	Male	Female
**Family subscale**		0.81	0.81	0.80	0.82	0.82	0.81
3. My family really tries to help me.	5.83±1.29						
4. I get the emotional help and support I need from my family.	5.34±1.46						
8. I can talk about my problems with my family.	5.03±1.32						
11. My family is willing to help me make decisions.	5.48±1.30						
**Friends subscale**		0.82	0.74	0.77	0.76	0.74	0.78
6. My friends really try to help me.	5.25±1.22						
7. I can count on my friends when things go wrong.	4.19±1.56						
9. I have friends with whom I can share my joys and sorrows.	5.37±1.26						
12. I can talk about my problems with my friends.	4.78±1.33						
**Significant Others subscale**							
1. There is a special person who is around when I am in need.	5.48±1.28	0.75	0.83	0.79	0.82	0.83	0.79
2. There is a special person with whom I can share my joys and sorrows.	5.48±1.15						
5. I have a special person who is a real source of comfort to me.	5.76±1.24						
10. There is a special person in my life who cares about my feelings.	5.55±1.37						

SD, standard deviation.

Republished from [13] under a CC BY license, with permission from Gregory D. Zimet, PhD., original copyright [1988].

## Discussion

The present study is the first to investigate the psychometric properties of the MSPSS in adolescent survivors of a disaster in Indonesia. In this population, the three-factor structure of the MSPSS showed replicability. The version of the scale translated into Indonesian also demonstrated a very good construct validity and internal consistency reliability; therefore, it supported the factor structure of the original scale [13] and did other adolescent studies, such as of Hong Kong adolescents [51] and of urban, largely African-American adolescents [17]. The results of our study were dissimilar from the results from Chou [16], who found a two-factor structure, although both were tested in Asian adolescent populations. Ours also differed from findings of a two-factor structure from an adult population living in South Asia [24].

Moreover, the MG-CFA of the scale in the present study also demonstrated very good configural, metric, and scalar invariances across gender groups of adolescent disaster survivors. Results of the present study are similar to those of a study conducted by Cheng and Chang [51], which demonstrated that their initial three-level measurement of invariance across gender groups was equally valid for Hong Kong male and female adolescents. Our findings on the measurement invariance were also supported by another study [25], which investigated the MSPSS in samples of undergraduate men and women and reported that both the configural and metric tests were fully invariant, while its metric equivalence was partially invariant. Our study also obtained similar results from a recent study conducted in Chinese parents of children with cerebral palsy in which the MSPSS held the same constructs across gender groups [21]. Our findings described how the structure of the Indonesian version of the MSPSS measures similar constructs and item contents for both male and female adolescent disaster survivors. Therefore, in future studies, males and females can be put together without worrying about gender effects on the structure of the Indonesian version of the MSPSS.

In the present study, the lowest factor loading we obtained in the model was 0.49 (Friends subscale; item 7: ‘I can count on my friends when things go wrong.’). This might have been caused by how our respondents interpreted the item’s content of counting on their friends when they were experiencing a problem. In practice, the higher factor loading indicates that the item or indicator is highly associated with the factor, and vice versa. It connects the factor of theoretical interest with an empirical variable which attempts to measure the factor. Hence, the loading should be conceived as a validity coefficient if the factor indeed reflects the phenomenon of interest. Therefore, item 7 in our study was not that highly correlated with the Friends subscale. In the CFA, one needs to focus on the goodness-of-fit indexes because they reflect the construct validity. Since all of the indexes in our model met the recommended criteria, we did not need to delete any item. Additionally, for practical significance, a factor loading of either 0.45 with a sample size of 150, 0.40 with a sample size of 200, or 0.35 with a sample size of 250 is acceptable [52]. Tabachnick and Fidell [53] following the recommendations of Comrey and Lee [54] also described that a factor loading of 0.32 indicates poor, 0.45 is fair, 0.55 is good, 0.63 is very good, and 0.71 is excellent.

Regarding the reliability analysis, our statistical results supported that every construct in the scale being reliable. This was similar to results of a study of Chinese parents of children with cerebral palsy [21] and supported all three of the subscales achieving very good reliability values. The scale also demonstrated good internal consistency reliability with CR values of >0.7; therefore, the scale is considered a reliable and valid tool to measure social support as perceived by adolescent disaster survivors. Hair and Black [47] described how good CR values indicate that the items are reliable and have high correlations within the same construct. Our findings supported that the scale and its three subscales had excellent internal consistency reliability overall and across gender subgroups. The results are consistent with previous evaluations of the reliability of the MSPSS across diverse samples and are similar to other studies’ findings [17].

In this study, some values of CA and the CR were very close, and even most of the CR values were higher compared to CA. The CR is considered to produce a better estimate of true reliability than CA [55]. Although both of them indicated the consistency of the item measured the proposed construct, we employed both CA and the CR because CA assumes unidimensionality and indicates that the items are equally related to the construct (factor loadings are the same for all items). However, the CR does not assume this because it takes into consideration the factor loadings of every item. The structural equation modeling approach that we used in this study was empirically assessed, and it overcame some of the limiting assumptions of CA [56].

Although our present findings supported the original version of the scale, there were still some limitations that should be noted. First, we invited participation of Indonesian junior and senior high school students who had previously experienced a traumatic event; therefore, we might not be able to generalize whether the same factor structure and findings would be obtained in other adolescent populations, such as in normative or clinical settings. However, our findings contribute to understanding how social support might work and operate among individuals from different cultural backgrounds. Moreover, nonclinical samples can provide descriptive information on the MSPSS, as described [25]. Second, we used a cross-sectional research design; therefore, we were unable to confirm if the results would be consistent in different time periods. However, despite the limitations of the findings, our study contributes to the MSPSS literature. For example, our study employed measurement invariance through a multiple-group CFA which was one component used to determine the score validity evidence and evaluate the construct-irrelevant variance [37]. Building evidence to confirm the theoretical constructs and measurement invariance is important in establishing valid comparisons of MSPSS item responses and its subscales across specific groups [26].

## Conclusions

In summary, the present study demonstrated that the Indonesian version of the MSPSS has unique psychometric characteristics, and the original version with a three-factor structure is applicable to Indonesian adolescent disaster survivors. Measuring social support in a specific population may have different expectations, because the nature of social networks and cultural backgrounds also differ [57]. The availability of the Indonesian version of the MSPSS will be very useful in helping academicians and researchers select an ideal social support scale. We conclude by noting that our findings provide strong support for the use of the MSPSS as a multidimensional-construct instrument. We suggest that future investigations using this scale should be conducted to complete the characteristics and add to the variability of analyzing the scale.

## Supporting information

S1 Dataset (SAV)
